# Human-annotated dataset for social media sentiment analysis for Albanian language

**DOI:** 10.1016/j.dib.2022.108436

**Published:** 2022-07-02

**Authors:** Fatbardh Kadriu, Doruntina Murtezaj, Fatbardh Gashi, Lule Ahmedi, Arianit Kurti, Zenun Kastrati

**Affiliations:** a University of Prishtina, Prishtina 10000, Kosovo; b Linnaeus University, Växjö 351 95, Sweden

**Keywords:** Sentiment analysis, Machine/deep learning, Affective computing, NLP, Text classification

## Abstract

Social media was a heavily used platform by people in different countries to express their opinions about different crises, especially during the Covid-19 pandemics. This dataset is created through collecting people's comments in the news items on the official Facebook site of the National Institute of Public Health of Kosovo. The dataset contains a total of 10,132 comments that are human-annotated in the Albanian language as a low-resource language. The dataset was collected from March 12, 2020, and this coincides with the emergence of the first confirmed Covid-19 case in Kosovo until August 31, 2020, when the second wave started. Due to the scarcity of labeled data for low-resource languages, the dataset can be used by the research community in the field of machine learning, information retrieval, affective computing, as well as by the public agencies and decision makers.

**Specifications Table**

**Table:** 

Subject	Computer Science
Specific subject area	Machine Learning, Natural Language Processing, Text Classification, eLearning, Affective Computing, Sentiment Analysis, Opinion Mining
Type of data	Table in csv format
How data were acquired	Dataset was collected and created using Facebook comments gathered from the National Institute of Public Health of Kosovo (NIPHK).
Data format	Raw and filtered
Description of data collection	Facebook comments between March 12, 2020 - August 31, 2020 collected via www.commentexporter.com from the site of National Institute of Public Health of Kosovo (NIPHK). Furthermore, the data is annotated into positive, neutral and negative sentiment by three researchers.
Data source location	Facebook site of National Institute of Public Health of Kosovo.
Data accessibility	Repository name: Mendeley Data repositoryData identification number: 10.17632/bj2gyvkgvx.4Direct URL to data: https://data.mendeley.com/datasets/bj2gyvkgvx/4
Related research article	Kastrati Z, Ahmedi L, Kurti A, Kadriu F, Murtezaj D, Gashi F. A Deep Learning Sentiment Analyser for Social Media Comments in Low-Resource Languages. Electronics. 2021; 10(10):1133. https://doi.org/10.3390/electronics10101133

**Value of the Data**

• This dataset is useful for the research community for two reasons: (1) it is a dataset for sentiment analysis of social media comments in Albanian language that would push forward the research in the field of sentiment analysis for low-resource languages; (2) this dataset could serve as a standard benchmark for testing performance of the existing and new machine learning methods and techniques as it is curated and human annotated. By sharing the data, we also increase the transparency and research utilization through enabling the reproduction of the results [1].
• The research community in the fields of machine learning, information retrieval, affective computing, education can benefit from these data by using them in various research tasks such as: (multilingual) sentiment analysis, opinion mining, performance analysis of deep/machine learning models and techniques. By making these data open we join the global movement that is not only advancing science and scientific communication but also transforming modern society and how decisions are made [2].
• Another possible value of these data is that they could be used by public agencies and decision makers to prevent the distribution of fake news in social media during crisis situations such as the current Covid-19 pandemics. This becomes especially important in the emerging economies where the scientific infrastructure is not very developed [3]. Thus, these kinds of curated scientific datasets can contribute to policy making as well.

## 1 Data Description

Research on social media sentiment analysis for the English language has achieved significant results already [4], [5], [6], considered as critical for different online social platforms and natural languages towards identifying online discourse, like are reactions from different cultures to the Coronavirus actions taken by different countries. Work on other languages concerning social media sentiment analysis is also growing, such as on German [7], Swedish [8], Urdu [9] or multilingual social media posts [10].

On the other hand, sentiment analysis for the Albanian language stands behind even some other low resource languages, with only few works on sentiment analysis (opinion mining) [11,12], emotion detection [13] and hate speech [14]. As deficiency of an Albanian language larger corpus of data is what these works characterize, being a prerequisite to develop a high-performance sentiment classifier for opinion mining, the dataset presented in this article aims to exactly address that low resource drawback typical for low-resource languages. Only a very recent study [14] has contributed with the so-called Shaj dataset, an annotated Albanian dataset of size range similar to our dataset, with 11874 comments from various social media platforms, but annotated for hate and offensive speech.

The Dataset presented in this article comprises comments collected from the official Facebook page of the NIPHK Institute for a period of 6 months, from March 12 till August 31, 2020. On March 12, the first case of Covid-19 was confirmed in Kosovo. Comments were in Albanian language and are retrieved using a tool called Comment Exporter^1^. This work aims to identify and extract the opinions and attitudes of Kosovo citizens expressed on Facebook about the Covid-19 pandemics by manually annotating comments according to their sentiment, such as positive, negative, and neutral. This dataset is anonymized according to the Facebook Platform Policy for Developers [15] and contains a total of 10,132 comments along with 12 attributes. The names of all attributes of the dataset and their respective descriptions are presented in Table 1. The data discussed in this article are related to the research article entitled “A Deep Learning Sentiment Analyser for Social Media Comments in Low-Resource Languages” [16]. The dataset and its supplementary files are hosted in the Mendeley Data repository [17].

**Table 1:** Description of the attributes constituting the dataset.

Attribute	Description
Id	unique identification number for each comment
Comment	the content of the comment
Like	the number of Facebook reactions to the relevant comment
Comment's timestamp	the day, date and time of the comment
Post's timestamp	the day, date and time of the post to which the comment belongs
#Deaths	number of persons who have died due to the pandemic in the given day
#Infected	number of infected persons with Covid-19
#Healed	number of people that have recovered from Covid-19
Annot 1	annotation of the comment by the annotator 1
Annot 2	annotation of the comment by the annotator 2
Annot 3	annotation of the comment by the annotator 3
Final annotation	the final assessment for the sentiment of the comment

Each comment in the dataset is assessed regarding the sentiment by three researchers. Neutral comments are marked with 0, positive comments are marked with 1 and negative comments are marked with 2. The comment with the most engagement has 287 impressions including likes and emojis.

On average each news post in the National Institute of Public Health of Kosovo (NIPHK) Facebook site generated 59.25 comments. Table 2 shows examples of neutral, positive and negative comments annotated by the researchers.

**Table 2:** Examples of neutral, positive and negative sentiments [2].

Comment (English translation)	Sentiment
Do te thot Peja^a^ edhe sonte spaska asnje rast (It means that also tonight Peja does not have any new case)	Neutral (0)
Bravo ekipet e IKShP per punen e shkelqyeshme dhe perkushtimin! (Well done the NIPHK teams for the great job and dedication!)	Positive (1)
Keni kalu tash ne monotoni, te pa arsyshem jeni tash. (You have now turned into monotony, you are now unreasonable.)	Negative (2)

a Peja is a city in Kosovo.

**Fig. 1:**
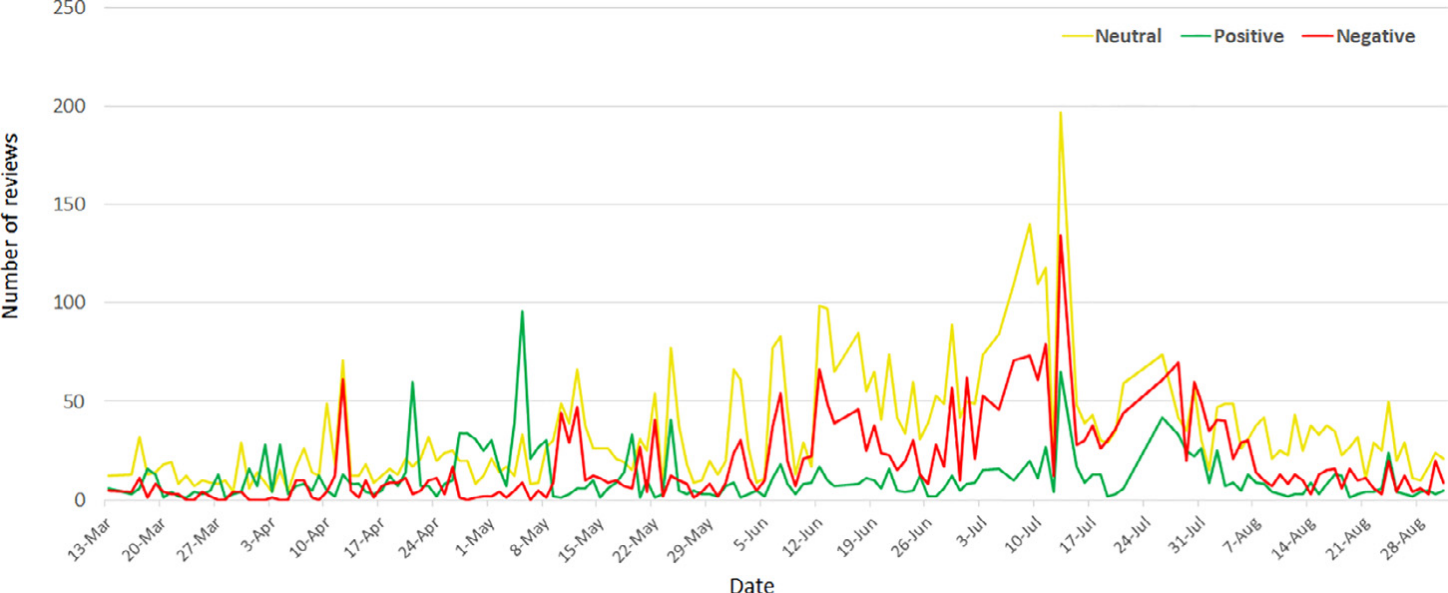
The daily trend of sentiments

The opinions of the people have changed over time, and this can be observed from Fig. 1 that shows the daily trend of the sentiments. This figure illustrates that the number of comments increases continuously starting from dozens of them, when the first cases of Covid-19 occurred and reached the peak during July. Then a noticeable decrease of the number of comments, in general, is shown during August. It should be noted that during all these dates the neutral sentiment dominates, compared to the negative and positive ones.

The number of comments for each sentiment class and their respective percentages are given in Table 3. The neutral comments dominate, whereas the positive comments are less represented.

**Table 3:** The number of comments for each sentiment class.

	Positive	Negative	Neutral
Number of comments	1771 (17.5%)	2910 (28.7%)	5451 (53.8%)

**Fig. 2:**
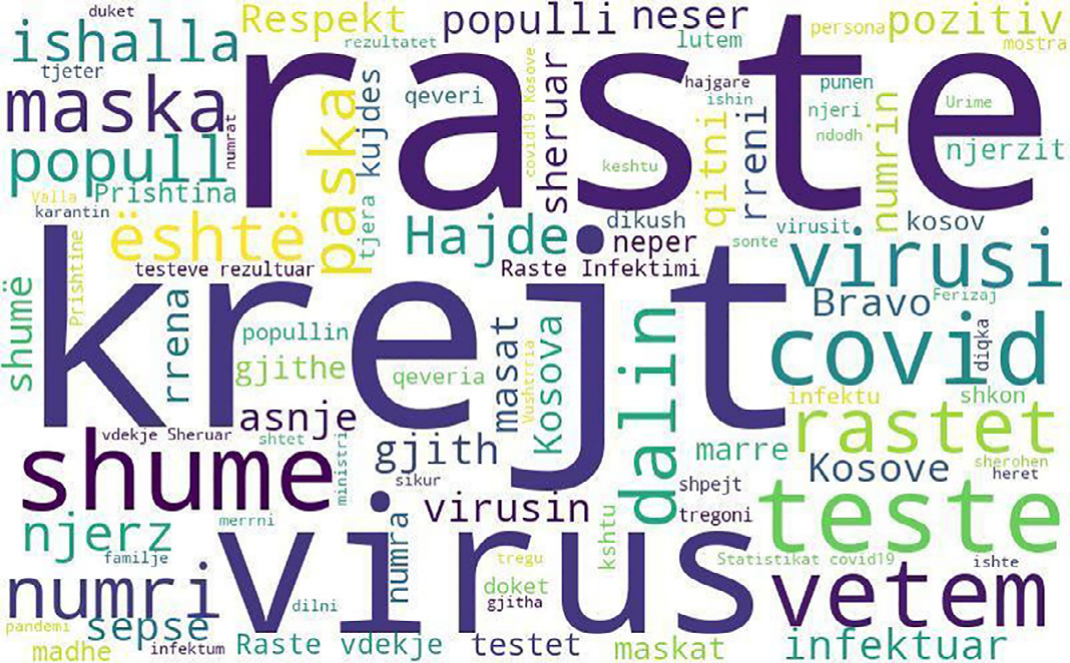
Word cloud for words in the dataset

The most frequent words in the dataset are shown in Fig. 2, generated by word cloud in Python. Only words with 5 or more characters are included.

The ten most frequent words occurring in the dataset are presented in Table 4. As text processing features such as stemming and lemmatization are not supported by tools for processing the Albanian language, then words with the same meaning can appear multiple times in different constructs (such as: raste - rastet, virus - virusi).

**Table 4:** Most frequent words in the dataset.

*No*.	Word	English translation	Frequency
1.	raste	cases	456
2.	krejt	all	401
3.	virus	virus	395
4.	shume	many	374
5.	test	test	353
6.	covid	covid	292
7.	vetem	only	277
8.	virusi	virus	252
9.	rastet	cases	226
10.	maska	masks	222

To assess the accuracy of the manually labeled data, we used a quality assurance method called Annotation Redundancy with Targeted Quality Assurance [18]. Three annotators have annotated the same data samples independently and an inter-annotator disagreement is calculated using Pearson correlation. Correlation values between the three annotators are shown in Table 5. The annotators 1 and 3 have the strongest agreement with a correlation of 0.62.

**Table 5:** The Pearson's correlation between the three annotators [2].

	Annotator 1	Annotator 2	Annotator 3
Annotator 1	1.00	0.57	0.62
Annotator 2	0.57	1.00	0.46
Annotator 3	0.62	0.46	1.00

In addition to Pearson correlation, we have calculated the reliability of the agreement between the annotators using Fleiss's Kappa statistical measure.

Table 6 shows the ranks which determine the type of agreements between annotators. Fleiss's Kappa coefficient for our three annotators is 0.60, which is considered a moderate agreement.

**Table 6:** Fleiss's kappa values.

k	Interpretation
<0	Poor agreement
0.01–0.20	Slight agreement
0.21–0.40	Fair agreement
0.41–0.60	Moderate agreement
0.61–0.80	Substantial agreement
0.81–1.00	Almost perfect agreement

The number of comments in each sentiment class, distributed over months is shown in Table 7. The maximum number of neutral and negative comments is reported in July. The highest number of positive comments is in May.

**Table 7:** Number of comments across months.

Sentiment\Month	March	April	May	June	July	August	Total
Neutral	228	565	816	1458	1477	907	5451
Positive	105	401	441	215	396	213	1771
Negative	54	205	349	749	1105	448	2910
Total	387	1171	1606	2422	2978	1568	10132

## 2 Experimental Design, Materials and Methods

**Fig. 3:**
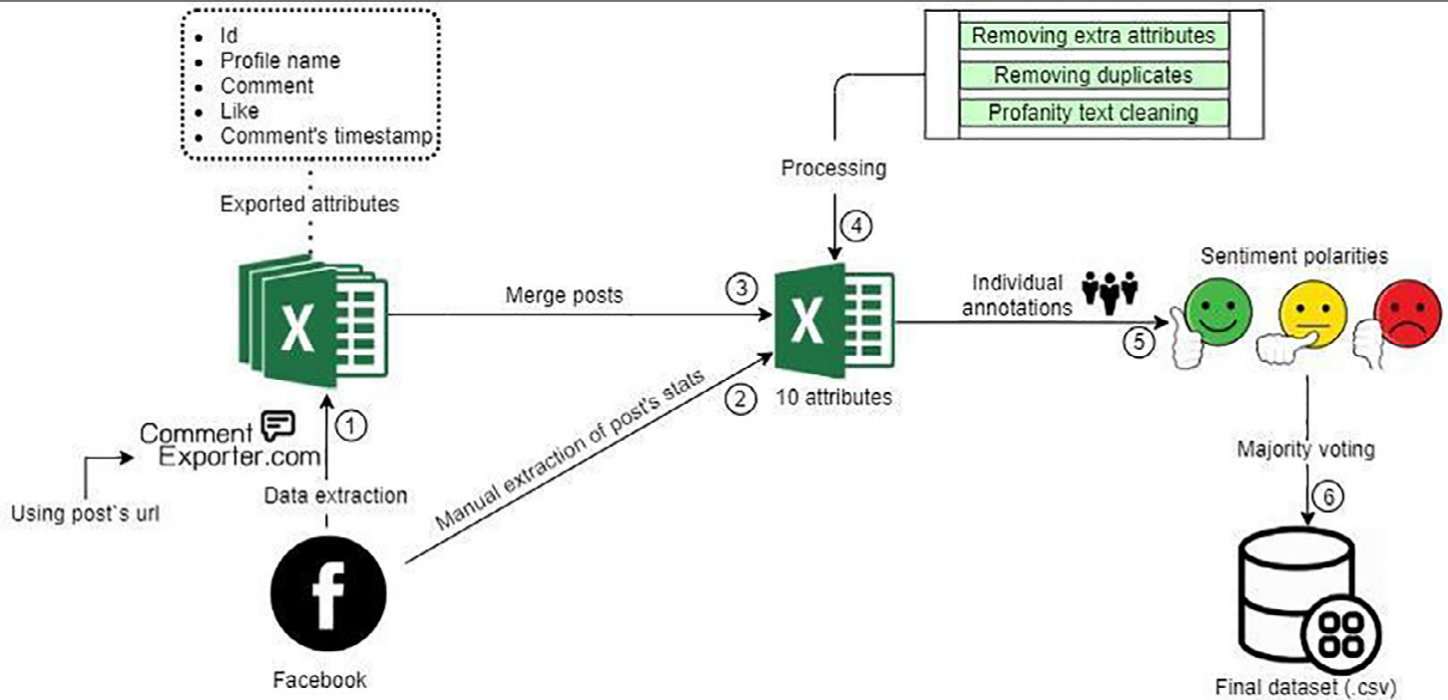
Workflow process for the creation of the dataset.

The workflow for creating the dataset is depicted in Fig. 3. The initial step is extraction of the comments from Facebook using the Comment Exporter tool. The entry point of the Comment Exporter tool is the Facebook post URL, while the output is an Excel file containing all the comments from the given post. In addition, the Excel file is enriched with four other attributes and metadata related to the Facebook post.

In the second step, five other attributes related to the Covid-19 statistics are added by reading the content of the Facebook post. The third step entails the merge of all Excel files for each Facebook post into a single Excel file. Next the aggregated data set has been processed by anonymizing and removing duplicates as well as profanity language. During the fifth step, three researchers have independently assessed the sentiment of the comments in the dataset. Assessment has been done using three sentiment polarities: positive, negative, and neutral. The final sentiment of each comment is assigned using a majority voting scheme. This step finalized our dataset as a CSV file.

The dataset covers comments from a sole social media platform, i.e. Facebook, over several-months timespan. It could in the future be extended to contain comments from other social media platforms like Twitter, Instagram, and so forth, spanning longer period of time. Future research using the dataset presented in this article might extend to other domains like detecting emotions or other multi-class text mining tasks (e.g., review of items in the scale 1 to 10) in the Albanian Language.

## Ethics Statements

Data has been collected according to the data owner terms of service. The dataset described in this article is completely anonymized and does not contain any personal data, and thus we are complying with the regulations provided by the platform owner. This project required that we balance our research design with the protection of personal data while aiming to generate new valuable knowledge for the greater societal good [19]. Consequently, we have chosen our approach to protect the confidentiality of the personal data completely and thoroughly, while still leveraging the anonymised data for generating research results of a higher societal value.

## CRediT authorship contribution statement

**Fatbardh Kadriu:** Methodology, Software, Writing – original draft. **Doruntina Murtezaj:** Software, Writing – original draft. **Fatbardh Gashi:** Software, Writing – original draft. **Lule Ahmedi:** Conceptualization, Methodology, Writing – original draft. **Arianit Kurti:** Conceptualization, Validation, Writing – original draft. **Zenun Kastrati:** Conceptualization, Validation, Writing – original draft, Supervision.

## Declaration of Competing Interest

The authors declare that they have no known competing financial interests or personal relationships which have or could be perceived to have influenced the work reported in this article.

## Appendix Supplementary materials

Image, application 1

## Footnotes

1 www.commentexporter.com.
